# *Sphingobium fuliginis* HC3: A Novel and Robust Isolated Biphenyl- and Polychlorinated Biphenyls-Degrading Bacterium without Dead-End Intermediates Accumulation

**DOI:** 10.1371/journal.pone.0122740

**Published:** 2015-04-13

**Authors:** Jinxing Hu, Mingrong Qian, Qian Zhang, Jinglan Cui, Chunna Yu, Xiaomei Su, Chaofeng Shen, Muhammad Z. Hashmi, Jiyan Shi

**Affiliations:** 1 Department of Environmental Engineering, College of Environmental and Resource Sciences, Zhejiang University, Hangzhou, China; 2 Institute of Quality and Standard on Agricultural Products, Zhejiang Academy of Agricultural Sciences, Hangzhou, China; 3 College of Life and Environmental Sciences, Hangzhou Normal University, Hangzhou, China; The University of Iowa, UNITED STATES

## Abstract

Biphenyl and polychlorinated biphenyls (PCBs) are typical environmental pollutants. However, these pollutants are hard to be totally mineralized by environmental microorganisms. One reason for this is the accumulation of dead-end intermediates during biphenyl and PCBs biodegradation, especially benzoate and chlorobenzoates (CBAs). Until now, only a few microorganisms have been reported to have the ability to completely mineralize biphenyl and PCBs. In this research, a novel bacterium HC3, which could degrade biphenyl and PCBs without dead-end intermediates accumulation, was isolated from PCBs-contaminated soil and identified as *Sphingobium fuliginis*. Benzoate and 3-chlorobenzoate (3-CBA) transformed from biphenyl and 3-chlorobiphenyl (3-CB) could be rapidly degraded by HC3. This strain has strong degradation ability of biphenyl, lower chlorinated (mono-, di- and tri-) PCBs as well as mono-CBAs, and the biphenyl/PCBs catabolic genes of HC3 are cloned on its plasmid. It could degrade 80.7% of 100 mg L ^−1^ biphenyl within 24 h and its biphenyl degradation ability could be enhanced by adding readily available carbon sources such as tryptone and yeast extract. As far as we know, HC3 is the first reported that can degrade biphenyl and 3-CB without accumulation of benzoate and 3-CBA in the genus *Sphingobium*, which indicates the bacterium has the potential to totally mineralize biphenyl/PCBs and might be a good candidate for restoring biphenyl/PCBs-polluted environments.

## Introduction

Biphenyl is a compound with two benzene rings [1]. It exists in natural gas, coal tar and crude oil. As an important industrial chemical, it has multiple applications, such as in the synthesis of plastics and crop protection products, in heat transfer fluids and as a parent compound of polychlorinated biphenyls (PCBs) [2,3]. It has been reported that biphenyl is a possible mutagen, and exposure to the compound can lead to toxic effects on nervous systems, can cause kidney disorders and can reduce hemoglobin levels [4,5]. The chlorinated derivatives of biphenyl, PCBs, are more toxic and cause serious effects on the nervous, immune and endocrine systems [6–9], in addition to causing cancer in animals [10]. Tanabe estimated that 0.37 million tons of PCBs had entered the environment [11]. Although the use of biphenyl and PCBs have been greatly reduced recently, they still remain in the environment because of their low bioavailability and stable structure. Thus, removal of these organic pollutants from contaminated environments are urgently needed to mitigate their damage to ecosystems.

Microorganisms appear to be more suitable for restoring contaminated environments because they are environmentally friendly and cost-effective [12]. A number of biphenyl-degrading bacteria, such as *Sphingobium yanoikuyae* B1, *Achromobacter* sp. BP3, *Dyella ginsengisoli* LA-4, *Rhodococcus* sp. R04 and so on, have been isolated from many environmental samples and deeply studied [13–18]. Even though these strains are able to degrade biphenyl, the degradation efficiency can still be limited by the strong hydrophobicity, low biodegradability and high biotoxicity of biphenyl [5,19]. Thus, improving biphenyl degradation by adding readily available carbon sources to enhance the growth rates and metabolic activity of these organisms is feasible. However, there are currently only a few studies that have explored this topic.

It is universally accepted that various PCB congeners can be cometabolized by biphenyl-degrading bacteria through the biphenyl catabolic pathway [17,20]. In fact, a complete biphenyl catabolic pathway includes two parts: biphenyl upper pathway (transformation of biphenyl/PCBs into benzoate/ chlorobenzoates and aliphatic acids) and biphenyl lower pathway (further mineralization of benzoate/ chlorobenzoates and aliphatic acid) [21]. Many biphenyl-degrading bacteria do not contain a complete biphenyl catabolic pathway [22], which might lead to accumulation of dead-end intermediates and cause potential damage during biphenyl/PCBs biodegradation. The most easily accumulated dead-end intermediates are benzoate and chlorobenzoates (CBAs) [23,24]. It has been proved benzoate and its derivatives can inhibit the growth of microorganisms [25,26] and cause health and environmental problems [27]. And the inhibition effects of CBAs on PCBs degradation have been also reported [23,28]. Thus, it is necessary to screen excellent strains which have the ability to degrade biphenyl and PCBs without dead-end intermediates accumulation, an area where related research is currently lacking. Although only few natural isolates able to metabolize both PCBs and CBAs have been described, many researchers have described bacterial recombinants able to express the enzymes for the upper and lower PCBs degradation pathways through genetic exchange and completely metabolize low chlorinated biphenyl [29,30]. However, they are easy to lose their ability to metabolize both substrates when they are grown under non-selective conditions [30,31]. Therefore, such study tried to find novel biphenyl- and PCBs-degrading bacteria without dead-end intermediates accumulation and provide new microorganism resources for in situ removal of persistent organic pollutants.

In our research, soil samples were collected from an electric and electronic waste (e-waste) recycling area in Taizhou (28.5605°N 121.3852°E, PCBs concentration 3.60 mg/kg), Zhejiang Province, China. Taizhou city has been involved in e-waste recycling for over 35 years [32] and farmland nearby the recycling areas has been severely polluted by PCBs from e-waste [33]. Our group was permitted by Taizhou Municipal People’s Government to investigate the soil contamination status of this area. A biphenyl- and PCBs-degrading bacterium, *S*. *fuliginis* HC3, was isolated and identified. Then, the effects of exogenous carbon sources on biphenyl degradation efficiency of HC3 were studied. After that, the variation trends of benzoate and 3-CBA during biphenyl and 3-CB degradation were investigated. Finally, the degradation ability of PCBs and CBAs of HC3 and the location of biphenyl/PCBs catabolic genes (termed *bph*) in HC3 were preliminarily studied.

## Materials and Methods

### Chemicals and samples

Biphenyl and benzoate were purchased from Sinopharm Chemical Reagent Co., Ltd (Shanghai, China). PCBs were purchased from Accustandard Co., Ltd (USA), including 2-chlorobiphenyl (2-CB), 3-chlorobiphenyl (3-CB), 2,4′-dichlorobiphenyl (2,4′-DCB), 3,3′-dichlorobiphenyl (3,3′-DCB), 2,4,4′-trichlorobiphenyl (2,4,4′-TrCB), 2,4′,5-trichlorobiphenyl (2,4′,5-TrCB), 2,2′,3,3′-tetrachlorobiphenyl (2,2′,3,3′-TeCB), 2,2′,4,5′-tetrachlorobiphenyl (2,2′,4,5′-TeCB), 2,3′,4′,5-tetrachlorobiphenyl (2,3′,4′,5-TeCB) and 2,2′,4,4′,5,5′-hexachlorobiphenyl (2,2′,4,4′,5,5′-HCB). 2-chlorobenzoate (2-CBA), 3-chlorobenzoate (3-CBA) and 4-chlorobenzoate (4-CBA) were obtained from Aladdin Chemical Co., Ltd (Shanghai, China). A mixture of bis(trimethylsilyl)trifluoroacetamide (BSTFA) and trimethylchlorosilane (TMCS) (99:1, v/v), obtained from Sigma-Aldrich (Beijing, China), was used as the derivatization reagent. All other reagents and chemicals were of the highest purity commercially available. Soil samples were collected from the surface layer (0–30 cm) of a long-term e-waste recycling area in Taizhou, Zhejiang Province, China.

### Media

The composition of the mineral salts medium (MSM, g L^-1^) was as follows: KH_2_PO_4_ 1.0, K_2_HPO_4_·3H_2_O 3.0, MgSO_4_ 0.15, FeSO_4_ 0.01, CaCl_2_ 0.005, NaCl 1.0, (NH_4_)_2_SO_4_ 0.5, and trace elements solution 0.1% (v/v) at pH 7.2. The trace elements solution contained the following (g L^-1^): Na_2_Mo_4_·H_2_O 6.7, ZnSO_4_·5H_2_O 28.0, CuSO_4_·5H_2_O 2.0, H_3_BO_4_ 4.0, MnSO_4_·5H_2_O 4.0 and CoSO_4_·7H_2_O 4.7 at pH 7.2. The Luria-Bertani (LB) medium contained (g L^-1^): NaCl 10.0, tryptone 10.0 and yeast extract 5.0, pH 7.2. Both media were sterilized by autoclaving at 121°C for 20 min.

### Enrichment, isolation and identification of biphenyl-degrading bacteria

The soil samples (5 g) as well as MSM (100 mL) containing 200 mg L^-1^ biphenyl were placed in a flask and incubated on a shaker at 30°C and 180 rpm for 6 days. Then, 10 mL of enrichment culture was transferred into fresh media. After 6 generation times, the enrichment culture was diluted and spread on MSM agar plates and sprayed with biphenyl solution (dissolved in acetone, 10 g L^-1^) as a carbon source. The plates were incubated at 30°C, and colonies with yellow halos were selected and purified on LB agar plates. The yellow halos were caused by the formation of a kind of intermediate (2- hydroxy-6-oxo-6-phenylhex-2,4-dienoic acid, HOPDA) during biphenyl degradation.

A biphenyl-degrading bacterial strain HC3 isolated from the soil was identified using the Biolog GEN III system. In addition, the 16S rRNA gene of HC3 was amplified by PCR with a pair of forward and reverse primers: 27f (5′-AGA GTT TGA TCC TGG CTC AG-3′) and 1492r (5′-TAC CTT GTT ACG ACT T-3′) [34]. The 16S rRNA gene sequence and related sequences acquired from GenBank were aligned by BIOEDIT version 7.0 software. A phylogenetic tree was built by the neighbor-joining method [35] as implemented in MEGA version 4.0 software.

The sample of HC3 for scanning electron microscopy (SEM) was hardened by 2.5% glutaraldehyde solution for 12 h. Then the sample was dehydrated in a series of acetone solution (30%, 50%, 70%, 80%, 90%, 95% and 100%) for 30 min each. After that, the sample was suspended in isoamyl acetate for 15 min and dried by a critical point drier (Hitachi HCP-2) for 2 h. Finally, the sample was fixed and scanned under a SEM (Hitachi S-3000N).

### Biphenyl degradation and cell growth experiments

To obtain highly active cells, HC3 was grown in 30 mL of LB medium with 10 mg L^-1^ biphenyl at 30°C and 180 rpm on a shaker for 24 h. Then, the cells were collected by centrifugation at 8000 rpm for 5 min and washed twice with phosphate buffers (0.05 M, pH 7.2). After that, the cells were resuspended in MSM and adjusted to an OD_600_ of 1.0 for the following experiments.

The biodegradation of biphenyl by HC3 was conducted in 100 mL flasks at 30°C and 180 rpm. 2 mL of HC3 cells (OD_600_ 1.0) was separately inoculated into 18 mL of MSM with 10, 50, 100, 200, 500 and 1000 mg L^-1^ biphenyl. Residual biphenyl was extracted with the improved method of Hong et al. [14] at 24 h. The test samples were mixed with equal volumes of ethyl acetate, then moved to glass tubes, oscillated heavily on a vortex for 10 min and set statically for 30 min. The organic phases were dried using anhydrous sodium sulfate and measured by GC-MS.

To measure the growth of HC3 on biphenyl, 1 mL of HC3 cells (OD_600_ 1.0) was inoculated into 19 mL of MSM with 200 mg L^-1^ biphenyl. The flasks were then cultured at 30°C and 180 rpm. LB medium and the spread plate method were used to enumerate HC3 cell number every 12 h and residual biphenyl was extracted at the same time. Because biphenyl is not easy to utilize as a carbon source by microorganisms, readily available carbon sources were selected to study their influence on biphenyl degradation. In this study, 1 g L^-1^ glucose, sodium acetate, tryptone and yeast extract was added to MSM separately as carbon sources. 2 mL of HC3 cells (OD_600_ 1.0) was inoculated into 18 mL of adjusted MSM with 100 mg L^-1^ biphenyl. Both the culture conditions and the detection methods were as previously described.

### Identification and analysis of benzoate and 3-CBA by GC-MS

For benzoate analysis, 100 μL of biphenyl solution (dissolved in acetone, 300 mg L^-1^) was added to sterile glass tubes. After the acetone evaporated, 2 mL of HC3 cells (OD_600_ 1.0) was added to the tubes. Then these tubes were sealed and cultured at 30°C and 180 rpm for 0, 40, 100 and 220 min. For 3-CBA analysis, 100 μL of 3-CB solution (dissolved in acetone, 200 mg L^-1^) was added to sterile glass tubes. Then 2 mL of HC3 cells (OD_600_ 1.0) was added to the tubes after the acetone evaporated. These tubes were sealed and cultured at 30°C and 180 rpm for 0, 0.5, 1, 1.5, 2, 2.5, 3, 4 and 4.5 h. At each timepoint, the samples were taken out and acidified to pH 2.0 using HCl (3 M). Biphenyl, 3-CB and their intermediates were extracted using an equal volume of ethyl acetate 2 times. Then, the organic phases were combined together and dried using anhydrous sodium sulfate and N_2_ flow. After that, the extracts were dissolved in 100 μL of hexane and 100 μL of BSTFA-TMCS (99:1, v/v) at 60°C for 15 min. Finally, the samples were diluted with hexane to 1 mL and analyzed by GC-MS.

### PCBs and CBAs degradation experiments

Before 3 mL of MSM and 2 mL of HC3 cells (OD_600_ 1.0) were added to sterile glass tubes, 10 PCB congeners (dissolved in hexane) were added to these tubes to a total concentration of 70 mg L^-1^. In control groups, HC3 cells were inactivated twice by autoclaving at 121°C for 30 min. All of the tubes were sealed and cultured at 30°C and 180 rpm for 72 h. This treatment method was also adopted to CBAs degradation experiment (including 2-CBA, 3-CBA, 4-CBA and benzoate). PCBs were extracted with the method described by Tu et al. [36], while CBAs were extracted with the method described by Fave et al. [37].

### Plasmid curing in strain HC3

The plasmid in HC3 was cured by growth in LB medium with sodium dodecyl sulfonate (SDS, 0. 005%, w/v). The wild type strain of HC3 (OD_600_ 1.0) was inoculated in this medium and cultured at 30°C and 180 rpm for 24 h. After 3 generation times the culture was spread on LB agar plates and the colonies were selected to extract plasmid DNA by the alkaline lysis method [38]. For restriction enzyme digestion of plasmid DNA, 17 μL extracted plasmid DNA was incubated with 1 μL *Eco*R I/Hind III and 2 μL 10 × buffer at 37°C for 2 h. Plasmid DNA preparations were electrophoresed in 1.0% agarose gels at 4.0 V min^-1^ in 1 × TAE buffer for 1 h. The gel was analyzed in a Bio-Rad universal hood II (Bio-Rad Laboratories, Segrate, Italy).

To analyze the stability of HC3, the wild type strain of HC3 was cultured in LB medium overnight and spread on LB agar plates. Then the colonies in these plates were inoculated on new LB agar plates and sprayed with biphenyl solution. Unsuccessful formation of yellow halos around the new colonies in 24 h meant these colonies lost their ability to use biphenyl. Biphenyl, benzoate, 3-CB and 3-CBA were used to study the degradation capacity of a plasmid-cured strain of HC3. The degradation system was identical with that mentioned in the above section (Identification and analysis of benzoate and 3-CBA by GC-MS) and all samples were cultured at 30°C and 180 rpm for 12 h and analyzed by GC-MS.

### Analytical methods

Biphenyl was measured using an Agilent 7890A gas chromatography connected with an Agilent 5975C mass selective detector and a DB-5 capillary column (30 m × 0.25 mm, 0.25 μm). The initial column temperature was 80°C with a 1-min hold, a 25°C min^-1^ increase to 140°C, then an 8°C min^-1^ increase to 180°C and finally a 15°C min^-1^ increase to 280°C. The ion source, injector and detector temperatures were 230°C, 290°C and 280°C, respectively. Highly pure He was used as a carrier gas with a constant flow rate of 1.0 mL min^-1^. The injecting volume was 2 μL without splitting. The electron impact mode was 70 eV, and the mass scan scope ranged from 50 to 550 amu. The derivatization reagents-treated samples were analyzed by the same method.

PCBs were analyzed by an Agilent 7890A gas chromatography connected with ^63^Ni electron capture detector and a DB-5 capillary column (30 m × 0.25 mm, 0.25 μm). The GC programming was initial temperature 150°C, 1 min; ramped at 10°C min^-1^ to 175°C; isothermal for 3.5 min; then ramped at 1.5°C min^-1^ to a final temperature of 250°C; isothermal for 3 min. The injector and detector temperatures were 300°C and 325°C, respectively. The injecting volume was 1 μL without splitting. Highly pure N_2_ was used as the carrier gas with a constant flow rate of 1.0 mL min^-1^. Benzoate and CBAs were measured on an Agilent 1100 series HPLC system equipped with a Zorbax Eclipse XDB-C18 column (4.6 mm × 250 mm, 5 μm). The injecting volume was 20 μL. Methanol and 0.25% acetic acid (v/v) in a 58:42 ratio (v/v) were used as the mobile phase at a flow rate of 1.0 mL min^-1^. Peaks were detected by UV absorption at 235 nm.

### Data analysis

All of the statistical analysis was performed using SPSS version 16.0 software. One-way analysis of variance was used for statistical comparisons. The significance level was *P* < 0.05.

## Results

### Isolation and identification of *S*. *fuliginis* HC3

**Fig 1:**
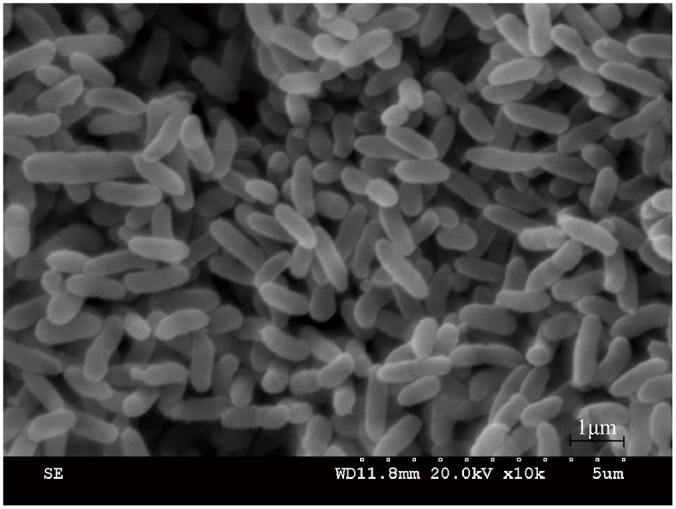
SEM image of HC3 at 10000 × magnification.

**Fig 2:**
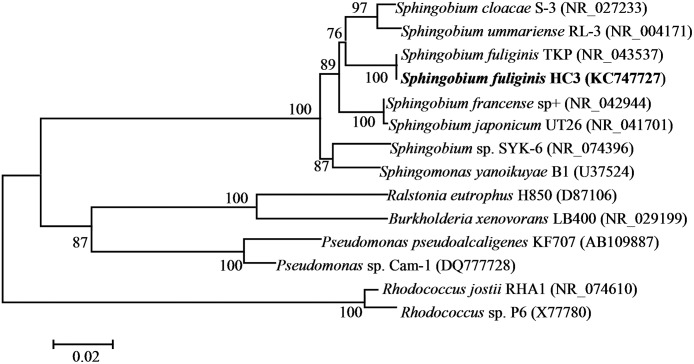
The neighbour-joining method cladogram showing a phylogenetic relationship between *Sphingobium fuliginis* HC3 and other related microorganisms in the genus *Sphingobium* and some deeply studied biphenyl/PCBs-degrading bacteria in other genera based on the 16S rDNA gene sequence analysis. Microorganisms’ names are followed by the accession numbers.

A biphenyl-degrading bacterium, HC3, was isolated from the soil samples and could form round, yellow-colored colonies with a smooth surface on LB agar plates. The bacterium was rod-shaped (Fig 1), gram-negative, oxidase positive, catalase positive, and nitrate-reduction positive, but the bacterium could not grow at 50°C and could not use starch, D-fructose and α-lactose as sole sources of carbon and energy. The partial 16S rRNA gene sequence (1391 bp) of HC3 was 99% similar to *Sphingobium fuliginis* TKP and 96% similar to *Sphingobium ummariense* RL-3 and *Sphingobium cloaca*e S-3 (Fig 2). Based on its 16S rRNA gene sequence, morphological features, and physiological and biochemical characteristics (S1 Table), HC3 was identified as *S*. *fuliginis* (accession number: KC747727).

### Degradation characteristics of biphenyl

**Fig 3:**
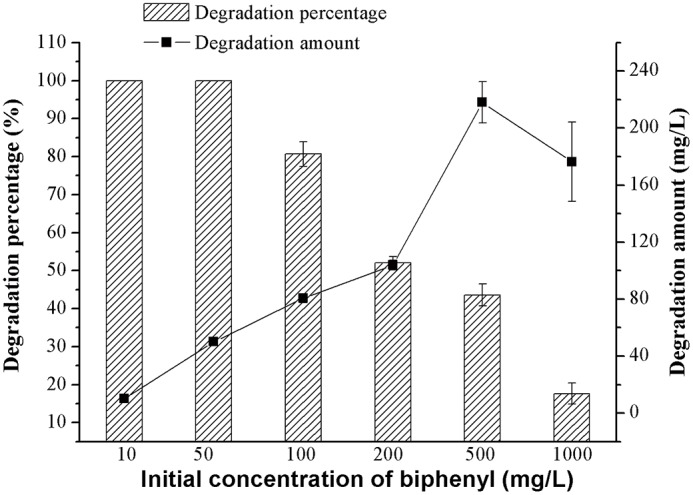
Degradation of various concentrations of biphenyl (10–1000 mg L−1) by HC3 in 24 hours. Inoculation amount was 10% (v/v). Error bars represent mean ± standard deviation (*n* = 3).

To study the biphenyl tolerance ability of HC3, a series of biphenyl concentrations from 10 to 1000 mg L^-1^ were adopted. The results are showed in Fig 3. With the increase of biphenyl concentration, the degradation percentage of biphenyl decreased, whereas the degradation amount of biphenyl presented the trend of first increasing and then decreasing. At an initial concentration of 500 mg L^-1^, 225.5 mg L^-1^ biphenyl was degraded within 24 h. Meanwhile, when the initial concentration of biphenyl was 1000 mg L^-1^, only 165.5 mg L^-1^ biphenyl was removed within a similar period of time. These results indicated that HC3 could tolerate at least 1000 mg L^-1^ biphenyl, but its biphenyl degradation ability could be inhibited at this concentration.

**Fig 4:**
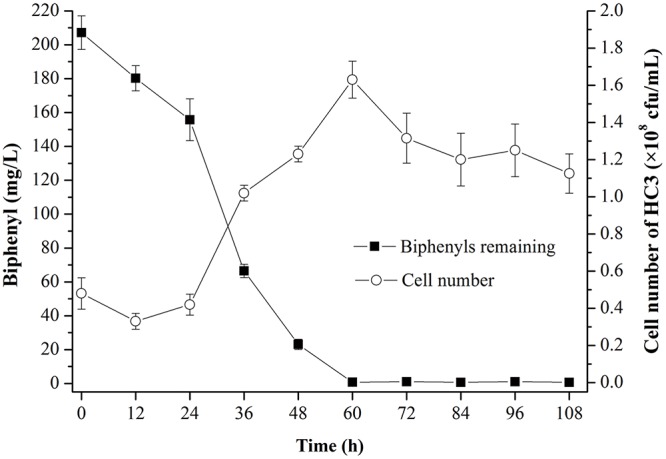
Time course of biphenyl degradation and cell growth of HC3.

**Fig 5:**
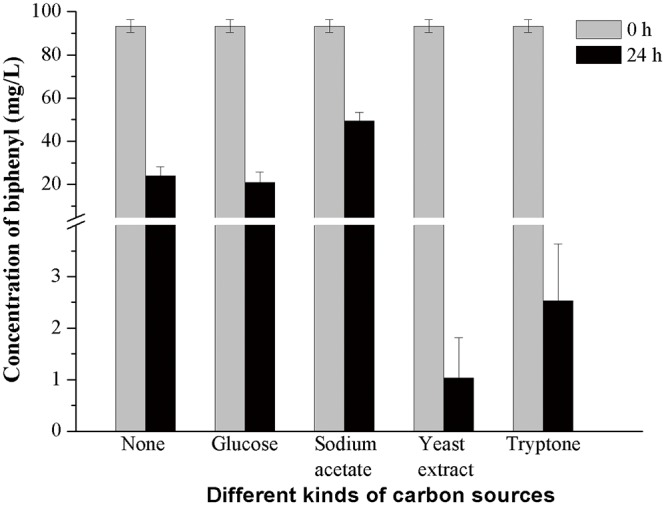
Effects of carbon sources on degradation of biphenyl (100 mg L^−1^) by HC3. Control group: active cells 10% (v/v), no additional carbon source. Other groups: active cells 10% (v/v), glucose, sodium acetate, yeast extract or tryptone 1 g L^-1^. Error bars represent mean ± standard deviation (*n* = 3).

The degradation of biphenyl and cell growth indicated that HC3 could utilize biphenyl as the sole carbon source (Fig 4). Four readily available carbon sources, namely glucose, sodium acetate, tryptone and yeast extract were chosen to study their influence on biphenyl degradation efficiency of HC3. The data indicated that biphenyl degradation efficiency was obviously improved by yeast extract and tryptone. Over 97% of biphenyl was degraded by HC3 within 24 h when yeast extract or tryptone was added to the media (Fig 5). Whereas in the other three groups, more than 20% of biphenyl still remained in the media at 24 h.

### Identification and analysis of metabolic intermediates: benzoate and 3-CBA

**Fig 6:**
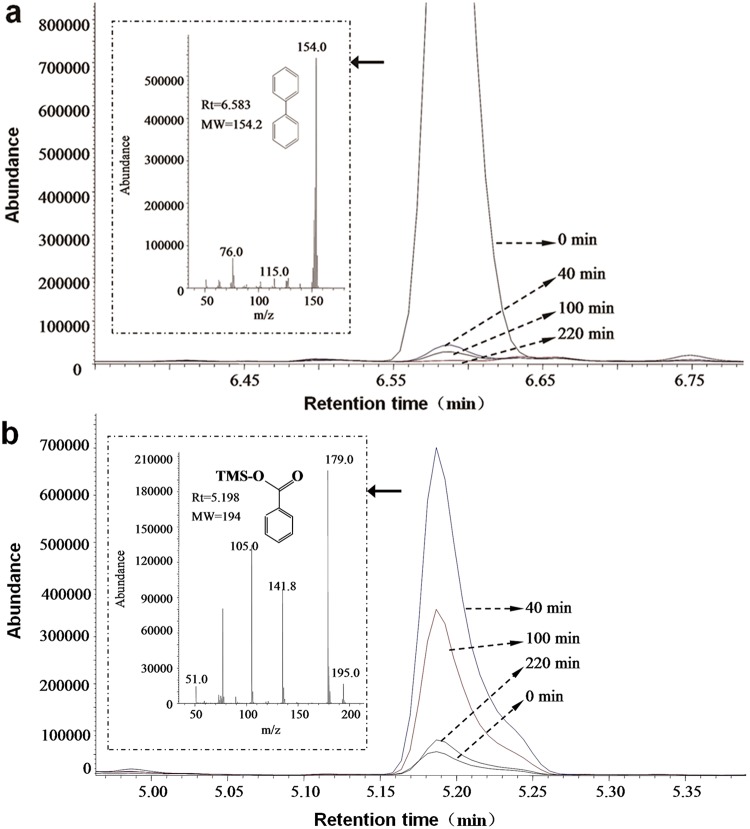
The variation trends of biphenyl and benzoate in biphenyl degradation system at 0–220 min, qualitatively analyzed by GC-MS. a. Biphenyl. b. Benzoate. Initial concentration of biphenyl was 15 mg L^-1^. The derivatization reagent was BSTFA-TMCS (99:1, v/v).

Biphenyl and its intermediates were extracted from the degradation system and analyzed by GC-MS after derivatization with BSTFA-TMCS. Biphenyl (molecular weight 154.2) was eluted at 6.583 min with a molecular-ion peak at *m/z* 154 (Fig 6a) and a new compound (molecule weight 194) was also eluted at 5.198 min with a molecular-ion peak at *m/z* 195 (Fig 6b). Both the retention time and the mass spectrogram of the new compound were identical with that of the derivative from benzoate (derivatization with BSTFA-TMCS), which indicated that the precursor of the new compound was benzoate. On the basis of the peak areas of biphenyl and benzoate, it was found that the content of benzoate initially increased and then decreased (Fig 6b) in pace with the degradation of biphenyl (Fig 6a), though the exact benzoate was not quantified.

**Fig 7:**
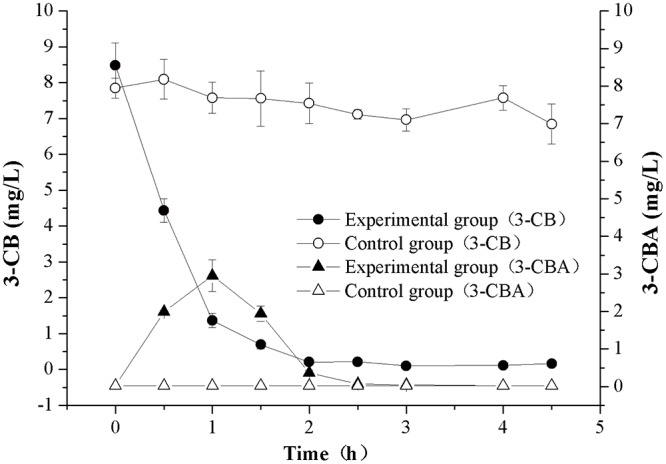
The variation trends of 3-CBA in 3-CB degradation system at 0–5 h, quantitatively analyzed by GC-MS. Initial concentration of 3-CB was 10 mg L^-1^. In control groups, HC3 cells were inactivated twice by autoclaving at 121°C for 30 min. The derivatization reagent was BSTFA-TMCS (99:1, v/v). Error bars represent mean ± standard deviation (*n* = 3).

When 3-CB was used as the degradation substrate of HC3, the production of 3-CBA was also found. GC-MS was used to identify and quantify 3-CB and 3-CBA in order to accurately analyze their variation trends. In the experimental group 3-CB was totally degraded in the initial 2 h (Fig 7). Meanwhile the concentration of 3-CBA increased from 0 mg L^-1^ to 2.94 mg L^-1^ in the initial 1 h and then decreased to 0 mg L^-1^ in the following 1 h. Therefore, biphenyl and 3-CB could be separately degraded into benzoate and 3-CBA and further transformed into other substances by HC3.

### Degradation characteristics of PCBs and CBAs

It is well known that various PCBs can be cometabolized by biphenyl-degrading bacteria in aerobic condition, especially PCB congeners with less than four chlorine substituents. The results of PCBs degradation by HC3 are presented in Table 1. After 3 days of biodegradation, mono-, di- and tri-PCBs were significantly degraded in comparing with the control group, while the degradation percentage of tetra- and hex-PCBs were insignificant. Table 2 shows the results of CBAs and benzoate degradation by HC3. 3-CBA, 4-CBA and benzoate were nearly totally degraded after 3 days of incubation. However, the degradation of 2-CBA was not remarkable.

**Table 1:** Degradation of 10 PCB congeners by HC3 in 72 hours.

PCB congener	Initial	Control group		Experimental group	
	Concentration (mg L^-1^)	Concentration (mg L^-1^)	Biodegradation (%)	Concentration (mg L^-1^)	Biodegradation (%)
2-CB	5.57±0.26a	3.44±0.40b	40.2±7.0	0.00±0.00c	100.0±0.0
3-CB	6.41±0.31a	5.96±0.41b	3.3±1.3	0.00±0.00c	100.0±0.0
2,4′-DCB	7.18±0.28a	6.37±0.24b	11.3±3.4	0.20±0.04c	97.2±0.5
3,3′-DCB	7.93±0.19a	7.36±0.16a	7.2±2.0	4.60±0.35b	42.0±4.4
2,4,4′-TrCB	7.43±0.05a	6.92±0.32a	6.9±4.4	5.03±0.53b	32.3±7.1
2,4′,5-TrCB	9.36±0.38a	7.56±0.32b	19.3±3.4	6.62±0.10c	29.3±1.0
2,2′,3,3′-TeCB	16.73±0.62a	14.98±0.72b	8.5±4.4	14.75±0.18b	9.9±1.1
2,2′,4,5′-TeCB	4.53±0.18a	3.82±0.23b	15.8±5.1	3.66±0.03b	19.3±0.2
2,3′,4′,5-TeCB	2.44±0.12a	2.28±0.09a	6.7±3.8	2.27±0.02a	7.1±1.0
2,2′,4,4′,5,5′-HCB	5.51±0.08a	4.97±0.04b	9.7±0.7	5.03±0.06b	8.7±1.1

Note: Control group, inactive cells 40% (v/v). Experimental group, active cells 40% (v/v). Date are presented as mean ± standard deviation (*n* = 3). Data followed by the same lowercase letter in each row are not significantly different (*P* > 0.05).

**Table 2:** Degradation of mono-CBAs and benzoate by HC3 in 72 hours.

Substrate	Initial	Control group		Experimental group	
	Concentration (mg L^-1^)	Concentration (mg L^-1^)	Biodegradation (%)	Concentration (mg L^-1^)	Biodegradation (%)
Benzoate	19.17±0.50a	17.70±0.37b	7.7±1.9	-0.06±0.10c	100.3±0.5
2-CBA	19.55±1.50a	19.12±1.66a	2.2±8.5	17.49±2.28a	5.3±4.3
3-CBA	13.78±0.69a	11.57±0.17b	16.0±1.3	0.09±0.00c	99.4±0.0
4-CBA	34.27±3.28a	28.06±1.05b	18.1±3.1	-0.19±0.41c	100.6±1.2

Note: Control group, inactive cells 40% (v/v). Experimental group, active cells 40% (v/v). Date are presented as mean ± standard deviation (*n* = 3). Data followed by the same lowercase letter in each row are not significantly different (*P* > 0.05).

### Plasmid curing

**Fig 8:**
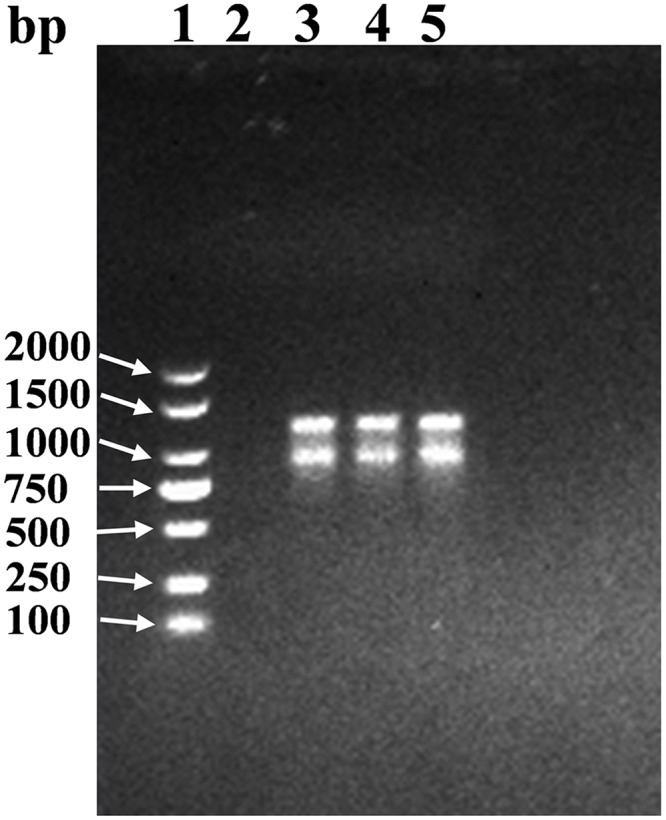
Agarose electrophoresis showing plasmid profile of HC3. Line 1, DL2000 plus DNA Landder Marker; Line 2, plasmid-free (SDS cured), Line 3, HC3 plasmid DNA (no digestion); Line 4, HC3 plasmid DNA (*Eco*R I digestion); Line 5, HC3 plasmid DNA (Hind III digestion).

Curing of the plasmid in HC3 was implemented with SDS. Compared with the wild type strain of HC3, the plasmid-cured strain completely lost its power to degrade biphenyl, 3-CB, benzoate and 3-CBA, which indicated that the *bph* genes of HC3 were cloned on its plasmid but not on its chromosomes (Table 3). Agarose gel electrophoresis showed HC3 might contain one plasmid, but the plasmid had no restriction enzyme sites for *Eco*R I and Hind III (Fig 8). And 6% of the colonies grown overnight in LB medium lost their capacity to degrade biphenyl, which meant that biphenyl degradation characteristic of HC3 was unstable.

**Table 3:** Degradation capacity of a wild type strain and a plasmid-cured strain of HC3.

Substrate	Initial	Control group	HC3 (plasmid-free)	HC3 (plasmid-containing)
	Concentration (mg L^-1^)	Biodegradation (%)	Biodegradation(%)	Biodegradation(%)
Biphenyl	9.45±0.85	6.2±2.5b	12.2±3.8b	94.0±2.2a
3-CB	8.49±0.51	14.1±5.5b	21.48±1.8b	78.1±1.0a
Benzoate	11.65±0.42	4.9±5.8b	4.6±2.0b	98.1±0.8a
3-CBA	10.68±0.44	2.4±4.0b	2.5±1.2b	99.9±0.2a

Note: Control group, inactive cells (OD_600_ 1.0). Experimental group, active cells (OD_600_ 1.0). Date are presented as mean ± standard deviation (*n* = 3). Data followed by the same lowercase letter in each row are not significantly different (*P* > 0.05).

## Discussion

*S*. *fuliginis* HC3 was isolated from PCBs-contaminated soil in an e-waste recycling region and is able to degrade biphenyl and PCBs. It has been observed that many species in the genus *Sphingobium*, such as *Sphingobium* sp. strain P2 [39], *Sphingobium amiense* sp. nov. strain YT^T^ [40], *Sphingobium fuliginis* sp. nov. strain TKP^T^ [41], *Sphingobium fuliginis* TIK-1 [42] and *Sphingobium yanoikuyae* B1 (formerly *Sphingomonas yanoikuyae* B1) [43], have the ability to degrade aromatic compounds, such as naphthalene, phenanthrene, nonylphenol, 4-tert-butylphenol, toluene and biphenyl. Among the many members of the genus *Sphingobium*, HC3 is the first discovered that is able to degrade biphenyl and PCBs without accumulation of benzoate and CBAs.

It has been reported that *Mycobacterium* sp. PYR-1 was able to degrade over 98% of 80 mg L^-1^ biphenyl within 72 h [44], whereas *Dyella ginsengisoli* LA-4 could degrade 95% of 100 mg L^-1^ biphenyl within 72 h [16] and *Achromobacter* sp. BP3 could totally degrade 50 mg L^-1^ biphenyl within 28 h [14]. In this study, HC3 could degrade 80.7% of 100 mg L^-1^ biphenyl within 24 h (Fig 3). However, when the concentration of biphenyl was over 500 mg L^-1^, the degradation capacity of HC3 could be inhibited, even though it could grow on 1000 mg L^-1^ biphenyl, which was similar to *Dyella ginsengisoli* LA-4 [45].

Because of the strong hydrophobicity, low biodegradability and high biotoxicity of recalcitrant organics, such as polycyclic aromatic hydrocarbons (PAHs), PCBs and biphenyl, the biodegradation efficiency of them can be greatly limited [5,19,46,47,48]. When biphenyl was used as the sole carbon source, the lag phase of HC3 lasted for 24 h (Fig 4). After the lag phase, the biphenyl degradation efficiency of HC3 began to increase in pace with the cell growth. This phenomenon has also been found by Chang et al.[49] and Adebusoye et al.[50]. Thus, biodegradation of these compounds could be possibly enhanced by stimulating the growth of microorganisms with readily available carbon sources such as pyruvate and glucose, which has been proven by Lee et al. [51] and Luo et al. [52]. In the current study, the biphenyl degradation efficiency of HC3 was obviously improved by tryptone and yeast extract (Fig 5). One possible reason is that tryptone and yeast extract contain abundant proteins, amino acids and vitamins, which serve as carbon sources, nitrogen sources, energy sources as well as growth factors for HC3.

Removal of biphenyl or PCBs does not mean these compounds can be totally mineralized to carbon dioxide and water. In fact, it is very easy to accumulate toxic intermediates during biphenyl and PCBs biodegradation because many biphenyl/PCBs-degrading bacteria do not contain a complete biphenyl catabolic pathway [22]. And the most easily accumulated dead-end intermediates are benzoate and CBAs, which have been reported to have negative effects on microorganisms growth and biphenyl/PCBs biodegradation [23,25,28]. Until now, only a few bacteria have been reported to have the ability to degrade biphenyl and benzoate or PCBs and CBAs simultaneously, including *Dyella ginsengisoli* LA-4 [16], *Burkholderia xenovorans* LB400 [53] and *Rhodococcus* sp. RHA1 [54]. LB400 and RHA1 have been proved to have relatively complete *bph* genes [55,56], which make these two strains have the potential to degrade a wide range of aromatic compounds. In our research, benzoate and 3-CBA transformed from biphenyl and 3-CB could be rapidly degraded by HC3 (Figs 6 and 7), which revealed that HC3 might contain a complete biphenyl catabolic pathway. Moreover, without the accumulation of benzoate or CBAs, the toxicity of these compounds on HC3 cells could be alleviated. This is one of the possible reasons why HC3 has high biphenyl degradation efficiency.

Although only a few bacteria have been reported to have the ability to degrade PCBs and CBAs simultaneously, many researchers have proved that bacterial recombinants could express the enzymes for the upper and lower PCBs degradation pathways through the assemblage of plasmid encoded genes and completely metabolize low chlorinated biphenyl [29,30]. However, plasmids are liable to be lost and bacteria will lose their ability to metabolize substrates when they are grown under non-selective conditions [30,31]. The *bph* genes of HC3 are cloned on its plasmid and thus its PCBs degradation capacity is unstable (Fig 8; Table 3). The loss rate of the ability to utilize special substrates per generation in non-selective media is 6% in HC3, 4 to 8% in *Acinetobacter* sp. strain P6 and *Arthrobacter* sp. strain M5 [57], 1% in *Alcaligenes eutrophus* JMP298 [31] and 8% in *Pseudomonas cepacia* CSV90 [58], which are possibly caused by the loss of plasmids in these strains. Since there are only a few bacteria like HC3 which is able to completely metabolize low chlorinated biphenyl, it is still necessary to screen this kinds of isolates from natural environments to enrich the bank of PCBs-degrading bacteria. In addition, wide-type degrading-bacteria can be used safely in soil remediation while the application of genetic engineering bacteria in natural environment is still restricted at present. By the way, it is well known that PCB degradation process is more efficient when performs by a mix population expressing both the upper and lower pathways [59,60]. Since a mix population usually contains different microorganisms, its metabolic pathways are more diverse and thus its substrate range is wider. In fact, the function of a mix population is also unstable. If the culture condition of a mix population changes, its original functions will change or lose [61,62]. So PCBs degradation capacity of both mix populations and pure cultures are still worth researching.

It has been reported that *bph* genes are not only present on plasmids but also present on bacterial chromosomes and transposons [63,64]. And the typical *bph* gene cluster is composed of *bphR1bphA1A2(orf3)bphA3A4BCX0X1X2X3D*, which is found in *Burkholderia* sp. strain LB400 [65–67] and *Pseudomonas pseudoalcaligenes* KF707 [68,69]. Furukawa et al. [1] compared the *bph* gene clusters of many bacteria and demonstrated that some are similar but some are very different on the basis of the structure of each gene and gene organization. These results indicated that certain *bph* gene clusters can transfer among soil bacteria and have evolved from a common ancestor. Thus, in order to determine the type of *bph* gene cluster in HC3, the differences of *bph* genes between HC3 and the ones that are already recorded in databank should be studied in subsequent research.

Actually, a major bottleneck in the PCB degradation pathway is the substrate range of the first enzyme (BphA) of the upper pathway. This enzyme is composed of a terminal dioxygenase containing a large subunit (encoded by *bphA1*) and a small subunit (encoded *bphA2*), and a ferredoxin (encoded by *bphA3*), and a ferredoxin reductase (encoded by *bphA4*) [68]. And it has been reported that the large subunit (BphA1) and the small subunit (BphA2) are responsible for the substrate specificity [63]. The amion acid sequences of BphA between *Pseudomonas pseudoalcaligenes* KF707 and *Burkholderia cepacia* LB400 are nearly identical (BphA1, 95.6%; BphA2, 99.5%; BphA3,100%; BphA4, 100%) [68,70], but the substrate ranges of these two strains are different [71,72]. In this research, the substrate range of HC3 is narrow compared to LB400, but HC3 has an excellent capacity to degrade biphenyl and low chlorinated biphenyl. Thus, it is significant to analyze BphA amion acid sequences of HC3. To obtain more information of BphA amion acid sequences will help researchers find out the relationship between BphA amion acid sequences and substrates specificity of PCBs-degrading bacteria.

In conclusion, an excellent biphenyl-degrading bacterial strain HC3, which could degrade 80.7% of 100 mg L^-1^ biphenyl within 24 h, was isolated from PCBs-contaminated soil. HC3 is also able to degrade benzoate, 3-CBA, 4-CBA, and PCBs with three or fewer chlorine atoms. The *bph* genes of HC3 are cloned on its plasmid. And the bacterium is the first reported that can degrade biphenyl and 3-CB without accumulation of benzoate and 3-CBA in the genus *Sphingobium*. All of these findings indicate that HC3 might contain relatively complete *bph* genes and has the potential to totally mineralize biphenyl and PCBs. Therefore, the bacterium might be a good candidate for restoring biphenyl/PCBs-contaminated environments.

## Supporting Information

S1 Table Test results of HC3 by Biolog GEN III. (DOC)

## References

[1] Furukawa K, Hikaru S, Masatoshi G. Biphenyl dioxygenases: functional versatilities and directed evolution. J Bacterio. 2004; 186: 5189–5196. 15292119 10.1128/JB.186.16.5189-5196.2004 PMC490896

[2] Hawley GG. Condensed chemical dictionary. 8th ed New York: Van Nostrand Reinhold; 1971.

[3] Weaver WC, Simmons PB, Thompson QE. Diphenyl and terphenyls In: Grayson M, Eckroth D, editors. Kirk-Othmer encyclopedia of chemical technology. New York: Wiley; 1979 pp. 789–793.

[4] Ambrose AM, Booth AN, DeEds F, Cox AJ Jr. A toxicological study of biphenyl, a citrus fungistat. J Food Sci. 1960; 25: 328–336.

[5] Boehncke A, Koennecker G, Mangelsdorf I, Wibbertmann A. Concise International Chemical Assessment Document 6. World Health Organization 1999 Available: http://www.inchem.org/documents/cicads/cicads/cicad06.htm.

[6] Aoki Y. Polychlorinated biphenyls, polychlorinated dibenzo-p-dioxins, and polychlorinated dibenzofurans as endocrine disrupters—what we have learned from Yusho disease. Environ Res. 2001; 86: 2–11. 11386736 10.1006/enrs.2001.4244

[7] Chen YQ, Adam A, Toure O, Dutta SK. Molecular evidence of genetic modification of *Sinorhizobium meliloti*: enhanced PCB bioremediation. J Ind Microbiol Biotechnol. 2005; 32: 561–566. 16208462 10.1007/s10295-005-0039-2

[8] Faroon O, Jones D, De Rosa C. Effects of polychlorinated biphenyls on the nervous system. Toxicol Ind Health. 2001; 16: 305–333. 10.1177/074823370001600708 11693948

[9] Man YB, Lopez BN, Wang HS, Leung AOW, Chow KL, Wong MH. Cancer risk assessment of polybrominated diphenyl ethers (PBDEs) and polychlorinated biphenyls (PCBs) in former agricultural soil of Hong Kong. J Hazard Mater. 2011; 195: 92–99. 10.1016/j.jhazmat.2011.08.010 21871716

[10] Mayes BA, Mc Connell EE, Neal BH, Burnner MJ, Hamilton SB, Peters AC, et al Comparative carcinogenicity in Sprague-Dawley rats of the polychlorinated biphenyl mixtures aroclors 1016, 1242, 1254, and 1260. Toxicol Sci. 1998; 41: 62–76. 9520342 10.1093/toxsci/41.1.62 PMC7107229

[11] Tanabe S. PCB problems in the future: foresight from current knowledge. Environ Pollut. 1988; 50: 5–28. 15092651 10.1016/0269-7491(88)90183-2

[12] Gan S, Lau EV, Ng HK. Remediation of soils contaminated with polycyclic aromatic hydrocarbons (PAHs). J Hazard Mater. 2009; 172: 532–549. 10.1016/j.jhazmat.2009.07.118 19700241

[13] Chadhain SMN, Moritz EM, Kim E, Zylstra GJ. Identification, cloning, and characterization of a multicomponent biphenyl dioxygenase from *Sphingobium yanoikuyae* B1. J Ind Microbiol Biot. 2007; 34: 605–613. 17647036 10.1007/s10295-007-0235-3

[14] Hong Q, Dong XJ, He LJ, Jiang X, Li SP. Isolation of a biphenyl-degrading bacterium, *Achromobacter* sp. BP3, and cloning of the *bph* gene cluster. Int Biodeterio Biodegrad. 2009; 63: 365–370.

[15] Lambo AJ, Patel TR. Isolation and characterization of a biphenyl-utilizing psychrotrophic bacterium, *Hydrogenophaga taeniospiralis* IA3-A, that cometabolize dichlorobiphenyls and polychlorinated biphenyl congeners in aroclor 1221. J Basic Microb. 2006; 46: 94–107. 16598832 10.1002/jobm.200510006

[16] Li A, Qu YY, Zhou JT, Guo M. Isolation and characteristics of a novel biphenyl-degrading bacterial strain, *Dyella ginsengisoli* LA-4. J Environ Sci-China. 2009; 21: 211–217. 19402424 10.1016/s1001-0742(08)62253-6

[17] Taguchi K, Motoyama M, Iida T, Kudo T. Polychlorinated biphenyl/biphenyl degrading gene clusters in *Rhodococcus* sp. K37, HA99, and TA431 are different from well-known *bph* gene clusters of *Rhodococci* . Biosci Biotechnol Biochem. 2007; 71: 1136–1144. 17485846 10.1271/bbb.60551

[18] Yang XQ, Sun Y, Qian SJ. Biodegradation of seven polychlorinated biphenyls by a newly isolated aerobic bacterium (*Rhodococcus* sp. R04). J Ind Microbiol Biot. 2004; 31: 415–420. 15365854 10.1007/s10295-004-0162-5

[19] Di Toro S, Zanaroli G, Fava F. Intensifiation of the aerobic bioremediation of an actual site soil historically contaminated by polychlorinated biphenyls (PCBs) through bioaugmentation with a non acclimated, complex source of microorganisms. Microb Cell Fact. 2006; 5: 1–10. 16549016 10.1186/1475-2859-5-11 PMC1456983

[20] Kohler HP, Kohler-Staub D, Focht DD. Cometabolism of polychlorinated biphenyls: enhanced transformation of aroclor 1254 by growing bacteria cells. Appl Environ Microb. 1988; 54: 1940–1945. 10.1128/aem.54.8.1940-1945.1988 PMC202783 3140725

[21] Aken VB, Correa PA, Schnoor JL. Phytoremediation of polychlorinated biphenyls: new trends and promises. Environ Sci Technol. 2010; 44: 2767–2776. 10.1021/es902514d 20384372 PMC3025541

[22] Pieper DH, Seeger M. Bacterial metabolism of polychlorinated biphenyls. J Mol Microbiol Biotechnol. 2008; 15: 121–138. 10.1159/000121325 18685266

[23] Adebusoye SA, Picardal FW, Ilori MO, Amund OO. Influence of chlorobenzoic acids on the growth and degradation potentials of PCB-degrading microorganisms. World J Microb Biot. 2008; 24: 1203–1208.

[24] Furukawa K, Tomizuka N, Kamibayashi A. Effect of chlorine substitution on the bacterial metabolism of various polychlorinated biphenyls. Appl Environ Microb. 1979; 38: 301–310. 117752 10.1128/aem.38.2.301-310.1979 PMC243481

[25] Brul S, Coote P. Preservation agents in foods: mode of action and microbial resistance mechanisms. Int J Food Microbiol. 1999; 50: 1–17. 10488839 10.1016/s0168-1605(99)00072-0

[26] Eklund T. Inhibition of microbial growth at different pH levels by benzoic and propionic acids and esters of *p*-hydroxybenzoic acid. Int J Food Microbiol. 1985; 2: 159–167.

[27] Keshavarz MH, Gharagheizi F, Shokrolahi A, Zakinejad S. Accurate prediction of the toxicity of benzoic acid compounds in mice via oral without using any computer codes. J Hazard Mater. 2012; 237–238: 78–101. 10.1016/j.jhazmat.2012.07.048 22959133

[28] Stratford J, Wright M, Reineke W, Mokross H, Havel J, Knowles C, et al Influence of chlorobenzoates on the utilization of chlorobiphenyls and chlorobenzoates mixtures by chlorobiphenyl/chlorobenzoate-mineralising hybird bacterial strains. Arch Microbiol. 1996; 165: 213–218. 8599540 10.1007/BF01692864

[29] Focht DD, Searles DB, Koh SC. Genetic exchange in soil between introduced chlorobenzoate degraders and indigenous biphenyl degraders. Appl Environ Microbiol. 1996; 62: 3910–3913. 8837452 10.1128/aem.62.10.3910-3913.1996 PMC168206

[30] Potrawfke T, Löhnert TH, Timmis K, Wittich RM. Mineralization of low-chlorinated biphenyls by *Burkholderia* sp. strain LB400 and by a two-membered consortium upon directed interspecies transfer of chlorocatechol pathway genes. Appl Microbiol Biot. 1998; 50: 440–446.

[31] Don R, Weightman A, Knackmuss H, Timmis K. Transposon mutagenesis and cloning analysis of the pathways for degradation of 2,4-dichlorophenoxyacetic acid and 3-chlorobenzoate in *Alcaligenes eutrophus* JMP134 (pJP4). J Bacteriol. 1985; 161: 85–90. 2981813 10.1128/jb.161.1.85-90.1985 PMC214838

[32] Shen CF, Chen YX, Huang SB, Wang ZJ, Yu CN, Qiao M, et al Dioxin-like compounds in agricultural soils near e-waste recycling sites from Taizhou area, China: chemical and bioanalytical characterization. Environ Int. 2009; 35: 50–55. 10.1016/j.envint.2008.07.005 18757099

[33] Shen CF, Huang SB, Wang ZJ, Qiao M, Tang XJ, Yu CN, et al Identification of Ah receptor agonists in soil of e-waste recycling sites from Taizhou area in China. Environ Sci Technol. 2008; 42: 49–55. 18350874 10.1021/es071162z

[34] Heuer H, Krsek M, Baker P, Smalla K, Wellington EM. Analysis of actinomycete communities by specific amplification of genes encoding 16S rRNA and gel-electrophoretic separation in denaturing gradients. Appl Environ Microbiol. 1997; 63: 3233–3241. 9251210 10.1128/aem.63.8.3233-3241.1997 PMC168621

[35] Saitou N, Nei M. The neighbor-joining method: a new method for reconstructing phylogenetic tree. Mol Biol Evol. 1987; 4: 406–425. 3447015 10.1093/oxfordjournals.molbev.a040454

[36] Tu C, Teng Y, Luo YM, Li XH, Sun XH, Li ZG, et al Potential for biodegradation of polychlorinated biphenyls (PCBs) by *Sinorhizobium meliloti* . J Hazard Mater. 2011; 186: 1438–1444. 10.1016/j.jhazmat.2010.12.008 21195547

[37] Fava F, Gioia DD, Marchetti L. Cyclodextrin effects on the ex-situ bioremediation of a chronically polychlorobiphenyl-contaminated soil. Biotechnol Bioeng. 1998; 58: 345–355. 10099268 10.1002/(sici)1097-0290(19980520)58:4<345::aid-bit1>3.0.co;2-j

[38] Feliciello I, Chinali G. A modified alkaline lysis method for the preparation of highly purified plasmid DNA from *Escherichia Coli* . Anal Biochem. 1993; 212: 394–401. 8214582 10.1006/abio.1993.1346

[39] Pinyakong O, Habe H, Yoshida T, Nojiri H, Omori T. Identification of three novel salicylate 1-hydroxylases involved in the phenanthrene degradation of *Sphingobium* sp. strain P2. Biochem Bioph Res Co. 2003; 301: 350–357. 10.1016/s0006-291x(02)03036-x 12565867

[40] Ushiba Y, Takahara Y, Ohta H. *Sphingobium amiense* sp. nov., a novel nonylphenol-degrading bacterium isolated from a river sediment. Int J Syst Evol Micr. 2003; 53: 2045–2048. 14657143 10.1099/ijs.0.02581-0

[41] Prakash O, Lal R. Description of *Sphingobium fuliginis* sp. nov., a phenanthrene-degrading bacterium from a fly ash dumping site, and reclassification of *Sphingomonas cloacae* as *Sphingobium cloacae* comb. nov. Int J Syst Evol Micr. 2006; 56: 2147–2152. 16957112 10.1099/ijs.0.64080-0

[42] Toyama T, Momotan IN, Ogata Y, Miyamori Y, Inoue D, Sei K, et al Isolation and characterization of 4-tert-butylphenol-utilizing *Sphingobium fuliginis* strains from *Phragmites australis* rhizosphere sediment. Appl Environ Microbiol. 2010; 76: 6733–6740. 10.1128/AEM.00258-10 20802076 PMC2953011

[43] Zylstra GJ, Kim E. Aromatic hydrocarbon degradation by *Sphingomonas yanoikuyae* B1. J Ind Microbiol Biot. 1997; 19: 408–414. 10.1038/sj.jim.2900475 26601331

[44] Moody JD, Doerge DR, Freeman JP, Cerniglia C. Degradation of biphenyl by *Mycobacterium* sp. strain PYR-1. Appl Microbiol Biot. 2002; 58: 364–369. 11935189 10.1007/s00253-001-0878-3

[45] Li A, Qu YY, Zhou JT, Ma F. Characterization of a newly isolated biphenyl-degrading bacterium, *Dyella ginsengisoli* LA-4. Appl Biochem Biotech. 2009; 159: 687–695. 10.1007/s12010-008-8513-8 19156364

[46] Ortega-Calvo JJ, Tejeda-Agredano MC, Jimemez-Sanchez C, Congiu E, Sungthong R, Niqui-Arroyo JL, et al Is it possible to increase bioavailability but not environmental risk of PAHs in bioremediation? J Hazard Mater. 2013; 261: 733–745. 10.1016/j.jhazmat.2013.03.042 23583067

[47] Providenti MA, Lee H, Trevors JT. Selected factors limiting the microbial degradation of recalcitrant compounds. J Ind Microbiol Biot. 1993; 12: 379–395.

[48] Zhao LJ, Jia YH, Zhou JT, Chen JF. Dynamics of augmented soil system containing biphenyl with *Dyella ginsengisoli* LA-4. J Hazard Mater. 2010; 179: 729–734. 10.1016/j.jhazmat.2010.03.062 20381236

[49] Chang Y, Taguchi K, Choi D, Toyama T, Sawada K, Kikuchi S. Isolation of biphenyl and polychlorinated biphenyl-degrading bacteria and their degradation pathway. Appl Biochem Biotechnol. 2013; 170: 381–398. 10.1007/s12010-013-0191-5 23529656

[50] Adebusoye SA, Ilori MO, Picardal FW, Amund OO. Metabolism of chlorinated biphenyls: use of 3,3′-and 3,5-dichlorobiphenyl as sole sources of carbon by natural species of *Ralstonia* and *Pseudomonas* . Chemosphere. 2008; 70: 656–663. 17706746 10.1016/j.chemosphere.2007.06.079

[51] Lee K, Park JW, Ahn IS. Effect of additional carbon source on naphthalene biodegradation by *Pseudomonas putida* G7. J Hazard Mater. 2003; 105: 157–167. 14623425 10.1016/j.jhazmat.2003.08.005

[52] Luo W, D’Angelo EM, Coyne MS. Organic carbon effects on aerobic polychlorinated biphenyl removal and bacterial community composition in soils and sediments. Chemosphere. 2008; 70: 364–373. 17870145 10.1016/j.chemosphere.2007.07.022

[53] Denef VJ, Park J, Tsoi TV, Rouillard JM, Zhang H, Wibbenmeyer JA, et al Biphenyl and benzoate metabolism in a genomic context: outlining genome-wide metabolic networks in *Burkholderia xenovorans* LB400. Appl Environ Microbiol. 2004; 70: 4961–4970. 15294836 10.1128/AEM.70.8.4961-4970.2004 PMC492332

[54] Seto M, Kimbara K, Shimura M, Hatta T, Fukuda M, Yano K. A novel transformation of polychlorinated biphenyls by *Rhodococcus* sp. strain RHA1. Appl Environ Microbiol. 1995; 61: 3353–3358. 16535122 10.1128/aem.61.9.3353-3358.1995 PMC1388576

[55] Chain PS, Denef VJ, Konstantinidis KT, Vergez LM, Agulló L, Reyes VL, et al *Burkholderia xenovorans* LB400 harbors a multi-replicon, 9.73-Mbp genome shaped for versatility. Proc Natl Acad Sci USA. 2006; 103: 15280–15287. 17030797 10.1073/pnas.0606924103 PMC1622818

[56] McLeod MP, Warren RL, Hsiao WW, Araki N, Myhre M, Fernandes C, et al The complete genome of *Rhodococcus* sp. RHA1 provides insights into a catabolic powerhouse. Proc Natl Acad Sci USA. 2006; 103: 15582–15587. 17030794 10.1073/pnas.0607048103 PMC1622865

[57] Furukawa K, Chakrabarty A. Involvement of plasmids in total degradation of chlorinated biphenyls. Appl Environ Microbiol. 1982; 44: 619–626. 6814360 10.1128/aem.44.3.619-626.1982 PMC242067

[58] Bhat M, Tsuda M, Horiike K, Nozaki M, Vaidyanathan C, Nakazawa T. Identification and characterization of a new plasmid carrying genes for degradation of 2,4-dichlorophenoxyacetate from *Pseudomonas cepacia* CSV90. Appl Environ Microbiol. 1994; 60: 307–312. 7509586 10.1128/aem.60.1.307-312.1994 PMC201304

[59] Fava F, Di Gioia D, Cinti S, Marchetti L, Quattroni G. Degradation and dechlorination of low-chlorinated biphenyls by a three-membered bacterial co-culture. Appl Microbiol Biot. 1994; 41: 117–123. 10.1007/BF00578472 8785040

[60] Hickey W, Searles D, Focht D. Enhanced mineralization of polychlorinated biphenyls in soil inoculated with chlorobenzoate-degrading bacteria. Appl Environ Microbiol. 1993; 59: 1194–1200. 8476293 10.1128/aem.59.4.1194-1200.1993 PMC202260

[61] Zache G, Rehm HJ. Degradation of phenol by a coimmobilized entrapped mixed culture. Appl Microbiol Biot. 1989; 30: 426–432.

[62] Yuan S, Wei S, Chang B. Biodegradation of polycyclic aromatic hydrocarbons by a mixed culture. Chemosphere. 2000; 41: 1463–1468. 11057584 10.1016/s0045-6535(99)00522-6

[63] Furukawa K. Biochemical and genetic bases of microbial degradation of polychlorinated biphenyls (PCBs). J Gen Appl Microbiol. 2000; 46: 283–296. 12483570 10.2323/jgam.46.283

[64] Sakai M, Masai E, Asami H, Sugiyama K, Kimbara K, Fukuda M. Diversity of 2,3-dihydroxybiphenyl dioxygenase genes in a strong PCB degrader, *Rhodococcus* sp. strain RHA1. J Biosci Bioeng. 2002; 93: 421–427. 16233225 10.1016/s1389-1723(02)80078-0

[65] Hofer B, Backhaus S, Timmis KN. The biphenyl/polychlorinated biphenyl-degradation locus (*bph*) of *Pseudomonas* sp. LB400 encodes four additional metabolic enzymes. Gene. 1994; 144: 9–16. 8026764 10.1016/0378-1119(94)90196-1

[66] Mondello FJ. Cloning and expression in *Escherichia coli* of *Pseudomonas* strain LB400 genes encoding polychlorinated biphenyl degradation. J Bacteriol. 1989; 171: 1725–1732. 2493454 10.1128/jb.171.3.1725-1732.1989 PMC209804

[67] Seeger M, Timmis KN, Hofer B. Conversion of chlorobiphenyls into phenylhexadienoates and benzoates by the enzymes of the upper pathway for polychlorobiphenyl degradation encoded by the *bph* locus of *Pseudomonas* sp. strain LB400. Appl Environ Microbiol. 1995; 61: 2654–2658. 7618878 10.1128/aem.61.7.2654-2658.1995 PMC167538

[68] Taira K, Hirose J, Hayashida S, Furukawa K. Analysis of *bph* operon from the polychlorinated biphenyl-degrading strain of *Pseudomonas pseudoalcaligenes* KF707. J Biol Chem. 1992; 267: 4844–4853. 1537863

[69] Watanabe T, Fujihara H, Furukawa K. Characterization of the second LysR-type regulator in the biphenyl-catabolic gene cluster of *Pseudomonas pseudoalcaligenes* KF707. J Bacteriol. 2003; 185: 3575–3582. 12775695 10.1128/JB.185.12.3575-3582.2003 PMC156218

[70] Erickson BD, Mondello FJ. Nucleotide sequencing and transcriptional mapping of the genes encoding biphenyl dioxygenase, a multicomponent polychlorinated-biphenyl-degrading enzyme in *Pseudomonas* strain LB400. J Bacteriol. 1992; 174: 2903–2912. 1569021 10.1128/jb.174.9.2903-2912.1992 PMC205943

[71] Suenaga H, Watanabe T, Sato M, Furukawa K. Alteration of regiospecificity in biphenyl dioxygenase by active-site engineering. J Bacteriol. 2002; 184: 3682–3688. 12057964 10.1128/JB.184.13.3682-3688.2002 PMC135152

[72] Bedard DL, Unterman R, Bopp LH, Brennan MJ, Haberl ML, Johnson C. Rapid assay for screening and characterizing microorganisms for the ability to degrade polychlorinated biphenyls. Appl Environ Microbiol. 1986; 51: 761–768. 3085588 10.1128/aem.51.4.761-768.1986 PMC238961

