# Supplementary material for: Sphingobium fuliginis HC3: A Novel and Robust Isolated Biphenyl- and Polychlorinated Biphenyls-Degrading Bacterium without Dead-End Intermediates Accumulation
Source: PLoS One. 2015 Apr 13;10(4):e0122740. doi: 10.1371/journal.pone.0122740 (PMC4395236; doi:10.1371/journal.pone.0122740)
Supplement: S1 Table — (DOC) [file pone.0122740.s001.doc]

**S1 Table.** Test results of HC3 by Biolog GEN III.

| Test items | Results | Test items | Results | Test items | Results | Test items | Results |
| --- | --- | --- | --- | --- | --- | --- | --- |
| Negative control | - | a-D-Glucose | + | Gelatin | - | p-Hydroxyphenyl-acetic acid | - |
| Dextrin | + | D-Mannose | - | Glycyl-L-proline | + | Methyl pyruvate | + |
| D-Maltose | + | D-Fructose | - | L-Alamine | + | D-Methyl lactate | - |
| D-Trehalose | + | D-Galactose | + | L-Arginine | - | L-Lactic acid | - |
| D-Cellobiose | + | 3-Methyl-D-glucose | - | L- Aspartic acid | - | Citric acid | - |
| Gentiobiose | + | D-Fucose | + | L-Glutamic acid | + | α-Ketoglutaric acid | - |
| Sucrose | - | L-Fucose | - | L-Histidine | - | D-Malic acid | - |
| Turanose | - | L-Rhamnose | - | L-Pyroglutamic acid | - | L- Malic acid | - |
| Stachyose | - | Inosine | - | L- Serine | - | Bromosuccinic acid | - |
| Postive contral | + | 1% Sodium lactate | + | Lincomycin | + | Nalidixic acid | + |
| pH 6.0 | + | Fusidic acid | - | Guanidine hydrochloride | - | Lithium chloride | - |
| pH 5.0 | - | D-Serine | - | Sodium tetradecyl sulfate | - | Potassium tellurite | + |
| D-Raffinose | - | D-Sorbitol | - | Pectin | - | Tween 40 | - |
| α-D-Lactise | - | D-Mannitol | - | D-Galacturonic acid | - | γ-Aminobutyric acid | - |
| D-Melibiose | - | D-Arabitol | - | L-Galactono-1,4- lactone | - | α-Hydroxybutyric acid | - |
| β-Methyl-D- glucoside | - | Inositol | - | D-Gluconic acid | - | β-Hydroxy-D,L- butyric acid | + |
| D-Salicin | - | Glycerine | - | D-Glucuronic acid | - | α-Ketobutyric acid | + |
| N-Acetyo-D- glucosamine | - | D-Glucose-6-  phosphate | - | Glucuronamide | - | Acetoacetic acid | + |
| N-Acetyl-β-D- mannsamine Monohdrate | - | D- Fructose -6- phosphate | - | Mucic acid | - | Propionic acid | + |
| N-Acetyl-D- galactosamine | - | D-Aspartic acid | - | Quinic acid | - | Acetic acid | + |
| N-  Acetylneuraminic acid | - | D- Serine | - | Saccharic acid | - | Formic acid | - |
| 1% NaCl | + | Troleandomycin | + | Vancomycin | - | Aztreonam | + |
| 4% NaCl | - | Rifamycin SV | + | Tetrazolium violet | - | Sodium butyrate | - |
| 8% NaCl | - | Minocycline | - | Tetrazolium blue | - | Sodium bromate | - |

Note: +, positive; -, negative
